# Supplementary material for: Whole blood transcriptomics identifies subclasses of pediatric septic shock
Source: Crit Care. 2023 Dec 8;27:486. doi: 10.1186/s13054-023-04689-y (PMC10709863; doi:10.1186/s13054-023-04689-y)
Supplement: Supplementary file 1 — Additional file 1. Online Data Supplement. [file 13054_2023_4689_MOESM1_ESM.docx]

**ONLINE DATA SUPPLEMENT**

Jamie O Yang^1^, Matt S. Zinter^2^, Matteo Pellegrini^3^, Man Yee Wong^4^, Kinisha Gala^4^, Daniela Markovic^5^_,_ Brian Nadel^6^, Kerui Peng^6^, Nguyen Do^1^, Serghei Mangul^6,7^, Vinay M. Nadkarni, MD^8^, Aaron Karlsberg^6^, Dhrithi Deshpande^6^, Manish J. Butte^9^, Lisa Asaro^10^, Michael Agus^10^, Anil Sapru, MD, MAS^4^.

1. UCLA Department of Internal Medicine, David Geffen School of Medicine
2. UCSF Department of Pediatrics
3. UCLA Department of Molecular, Cell, and Developmental Biology
4. UCLA Department of Pediatrics, Division of Critical Care
5. UCLA Department of Medicine Statistics Core
6. USC Department of Clinical Pharmacy, USC Alfred E Mann School of Pharmacy and Pharmaceutical Sciences
7. USC Dornsife College of Letters, Arts and Sciences, Department of Quantitative and Computational Biology
8. University of Pennsylvania Department of Anesthesiology and Critical Care Medicine, Children's Hospital of Philadelphia, and Perelman School of Medicine
9. UCLA Department of Pediatrics, Division of Immunology, Allergy, and Rheumatology
10. Boston Children’s Hospital, Department of Pediatrics, Division of Medical Critical Care, Harvard Medical School
11. Department of Quantitative and Computational Biology, USC Dornsife College of Letters, Arts and Sciences

**Supplemental Methods**

Quantification of Gene Expression: Total RNA was extracted from 500 μL whole blood using PAXgene Blood RNA Kit IVD (Qiagen, Hilden, Germany) according to the manufacturer’s directions. Samples were eluted in an 80 μL elution buffer. The extracted samples were stored in -80°C after extraction. The RNA yield was measured by Qubit RNA HS assay (Thermo Fisher). RNA integrity was assessed on the Agilent TapeStation using RNA ScreenTape (Agilent Technologies). An RNA integrity number (RIN) was assigned to each sample by the TapeStation system and n=24 samples were removed due to low sample quality (n=8 from day 0, n=10 from day 2, and n=6 from day 4). cDNA libraries construction and sequencing were performed at Technology Center for Genomics and Bioinformatics at UCLA using the Nugen universal plus kit with polyA capture. Sequencing was performed using the Novaseq S4 system (Illumina) in two rounds using 150 nucleotide paired-end sequencing to a target depth of 40-50 million read pairs per sample.

Reference genome (hg19) and gene model annotation files were downloaded from the UCSC genome browser and gencode websites directly. Indexes of the reference genome were built using STAR v2.7.0e. The quality of the raw reads was assessed using FastQC v0.11.9. The adaptors were clipped using cutadapt v1.18 (4) requiring at least three bases to match (--min overlap 3) and removing processed reads shorter than 20 bases (--min length 20). RNA-Seq reads were mapped to the NCBI v37 H. sapiens reference genome using STAR (v2.4.2a; with default option mismatch = 10). Bam files were sorted and indexed using SAMtools v1.9. Mapping statistics from the BAM files were acquired through Samtools flagstat v2.7.0e. Only uniquely aligned reads were used for downstream quantification and analysis. The percentage of reads marked as PCR duplicates, duplication rate was computed using Picard tools v2.13.2. HTSeq v0.13.5 was used to quantify gene expression. Sequencing was done in two batches with 241 samples sequenced in the first batch and sequenced in the second.

Expression of a total of 57,820 genes were quantified. For quality control, we removed genes that were not present in any of the patients, leaving 33,550 genes left. Then, we removed genes that were present in less than 50% of patients, leaving 20,010 genes left. Finally, we checked that all patients had counts in greater than 50% of the genes and that all patients had greater than 90,000 protein coding transcripts. No patients were removed from the analysis.

*ImRep Analysis of T cell Repertoire*

ImRep is a method to effectively and accurately profile T cell receptor (TCR) and immunoglobulin (Ig) repertoire from RNA-Seq data, including TCR clonotypes and V(D)J recombinations. ImRep uses both reads that are mapped to TCR or Ig genes as well as the unmapped reads to construct complementarity determining region 3 (CDR3) sequences. CDR3 is the most variable sequence region in the TCR encoded by the junctional region between V and J or D and J genes; and therefore can be used to determine the clonality and diversity of a T cell population. Subsequently, ImRep uses the Clustering Affinity Search Technique (CAST) technique to cluster assembled CDR3 sequences to reduce sequencing errors.
