# Supplementary material for: Whole blood transcriptomics identifies subclasses of pediatric septic shock
Source: Crit Care. 2023 Dec 8;27:486. doi: 10.1186/s13054-023-04689-y (PMC10709863; doi:10.1186/s13054-023-04689-y)
Supplement: Supplementary file 4 — Additional file 4. Supplemental Tables. [file 13054_2023_4689_MOESM4_ESM.docx]

**ONLINE DATA SUPPLEMENT TABLES**

Table E1: Pathway Enrichment of Differentially Expressed Genes between Septic Shock Cases and Controls

|  | **ID** | **Pathway Term** | **Differentially Expressed Genes Involved** | **Adjusted p value*** |
| --- | --- | --- | --- | --- |
| Upregulated Pathways in Septic Shock Cases | GO:0002283 | Neutrophil Activation Involved in Immune Response | SERPINB10, GPI, CD63, SIGLEC9, RAB3A, STXBP2, SLC2A3, RETN, PLAC8, TRPM2, HK3, S100A12, PGM2, STOM, QSOX1, ENPP4, GYG1, CTSD, CTSC, LAIR1, CD177, CTSA, VAT1, SERPINB1, AOC1, ATP8B4, GSN, FCER1G, ANXA3, AMPD3, OLFM4, MMP8, MMP9, CKAP4, OSCAR, NFASC, CLEC4D, PKM, VNN1, SLPI, TCN1, LCN2, HPSE, S100A9, S100A8, CD44 | 7.19 E -11 |
|  | GO:0002446 | Neutrophil Mediated Immunity |  | 7.19 E -11 |
|  | GO:0043312 | Neutrophil Degranulation |  | 7.19 E -11 |
|  | GO:0014070 | Response to Organic Cyclic Compound | GGT5, DHFR, TRPM2, ANXA1, TIPARP, CYP1B1, ALPL, GGT1, TNF | 1.64 E -2 |
|  | KEGG | Glutathione Metabolism | GGT5, G6PD, GPX3, GSTOI, CHAC1, OPLAH, GGT1, PGD, GCLM | 4.42 E -3 |
| Downregulated Pathways in Septic Shock Cases | GO:0048003 | Antigen Processing and Presentation of Lipid Antigen via MHC Class Ib | CD1E, CD1D, CD1C, CD1A | 1.40 E -4 |
|  | GO:0048006 | Antigen Processing and Presentation, endogenous lipid antigen via MHC Class Ib | CD1E, CD1D, CD1C, CD1A | 1.40 E -4 |
|  | GO: 0048007 | Antigen processing and presentation, exogenous lipid antigen via MHC Class Ib | CD1E, CD1D, CD1C, CD1A | 1.40 E -4 |
|  | GO:0001914 | Regulation of T cell mediated cytotoxicity | CD1E, KLRD1, CD1D, KLRC1, CD1C, CD1A | 1.74 E -3 |
|  | GO:0071396 | Cellular Response to Lipid | CD86, PID1, IRS1, ABCB4, CD180, GPBAR1, LPL, AHR, PTK7, TNFSF4, PDK4, SPP1, PF4V1 | 1.19 E -2 |
|  | GO: 0001912 | Positive Regulation of Leukocyte Mediated Cytotoxicity | KLRC2, CD1E, KLDR1, CD1D, CD1C, CD1A | 0.012 |
|  | GO:0071398 | Cellular Response to Fatty Acid | PID1, IRS1, PDK4, LPL | 0.022 |
|  | GO:0050776 | Regulation of Immune Response | TNFSF4, KLRF1, SH2D1B, KLRD1, KIR3DL1, AHR, CD1D, KLRC1, CD1C, CD1A, CD200 | 0.022 |
|  | GO:0070542 | Response to Fatty Acid | PID1, IRS1, PDK4, LPL | 0.030 |
|  | GO:0002711 | Positive Regulation of T cell Mediated Immunity | TNFSF4, CD1E, CD1D, CD1C, CD1A | 0.033 |

* The adjusted p-values are computed by using the Benjamini-Hochberg method for correction for multiple hypotheses testing. All Gene Ontology (GO) and KEGG pathways with an adjusted p value <0.05 are listed.

Table E2: Pathway Enrichment of Differentially Expressed Genes between Septic Shock Subclasses

|  | **ID** | **Pathway Term** | **Differentially Expressed Genes Involved** | **Adjusted p value*** |
| --- | --- | --- | --- | --- |
| Upregulated Pathways in Subclass 1 compared to Subclass 2 | GO:0002283 | Neutrophil Activation Involved in Immune Response | CDA, GMFG, ABCA13, MPO, PSMD6, LAMP1, PSMD1, SIRPA, CPNE3, ENPP4, CAPN1, OPSTF1, PGLYRP1, GYG1, CD177, NHLRC3, ATP11B, CYBA, OLFM4, OSCAR, CLEC4D, RAB31, DOK3, PRTN3, S100A9, ARL8A, S100A8, VCP, STXBP2, FPR1, PLD1, GNS, PLAC8, CNN2, NCSTN, CYB5R8, ALDH3B1, S100A12, NCKAP1L, RAB6A, ELANE, GSFMD, CTSA, VAT1, ATP8B4, TRAPPC1, RAB27A, NBEAL2, DNAJC13, ARPC5, PRDX6, BST1, VNN1, TCN1, TOLLIP, LCN2, CYSTM1, DEGS1, GPI, SERPINA1, SIGLEC9, ITGAM, SLC2A3, PYGL, FCAR, HK3, PGM2, CTSG, QSOX1, CTSD, PGM1, CAP1, ACTR2, SERPINB1, DBNL, FCER1G, DYNLT1, ANXA3, PGAM1, RHOG, AMPD3, MMP8, MMP9, CKAP4, FGR, PKM, DNAJC5, BPI, ALDOA, CD44, LTF, SERPINB10, CD63, GRN, MVP, RETN, AGPAT2, NRAS, ALOX5, STOM, CD59, CD55, LAIR1, AOC1, GSN, GCA, AGL, MAPK14, SVIP, VAPA, P2RX1, SLCO4C1, S100P | 2.29E-31 |
|  | GO:0002446 | Neutrophil Mediated Immunity |  | 2.80E-31 |
|  | GO:0043312 | Neutrophil Degranulation |  | 2.01E-31 |
|  | GO:0061024 | Membrane organization | GLTP,NAPA,HIP1,SAR1B,ARPC1A,DYSF,ARRB2,MOSPD3,ACTB,TOR1AIP1,ACTG1,SNX3,RIMS1,SYNGR2,UBC,CHP1,LDLR,CEP55,AGFG1,VTI1B,SH3GLB1,ACTR3,ACTR2,TOR1A,GCA,MYOF,ARPC5,TOR1B,SGCZ,VAPA,CHMP2B,CHMP4C,CHMP2A,CHMP4B,VAMP4,CFTR,HBEGF,VAMP3 | 2.21 E-4 |
|  | GO:0006096 | Glycolytic Process | PFKFB2;GPI;HK3;LDHA;PKM;PGAM1;PGK1;ENO1;ALDOA;GAPDH;PGM1 | 6.17 E -4 |
|  | GO:0009250 | Glucan Biosynthetic Process | GYS1;UGP2;GBE1;PGM2;GYG1;PGM2L1;PGM1 | 2.46 E -3 |
|  | GO:0005978 | Glycogen Biosynthetic Process | GYS1;UGP2;GBE1;PGM2;GYG1;PGM2L1;PGM1 | 2.46 E -3 |
|  | GO:0006090 | Pyruvate Metabolic Process | PFKFB2;GPI;PGAM1;ENO1;HK3;LDHA;PKM;PGK1;ME2;ALDOA;SLC16A3;GAPDH;PGM1 | 0.013 |
|  | GO:0016052 | Carbohydrate Catabolic Process | PFKFB2;GPI;HK3;LDHA;PKM;PGAM1;PGK1;ENO1;ALDOA;GAPDH;PGM1 | 0.014 |
|  | GO:0043174 | Nucleoside Salvage | CDA;TK2;UPP2;HPRT1;TK1;UPP1 | 0.015 |
|  | GO:0050727 | regulation of inflammatory response | GRN;GBA;IFI35;ADAMTS12;METRNL;USP18;LILRA5;SBNO2;HYAL2;ALOX5;S100A12;SIRPA;VPS35;GGT1;ELANE;IL10;ANXA1;OSM;NMI;MMP8;MMP9;BST1;HCK;NLRP12;PIK3AP1;S100A9;S100A8;PTGES;VAMP3 | 0.015 |
|  | GO:0050764 | regulation of phagocytosis | FCER1G;DYSF;RAB27A;CYBA;OLFM4;MERTK;FGR;SNX3;HCK;PLSCR1;SYT11;SIRPA;PRTN3 | 0.015 |
|  | GO:0022411 | cellular component disassembly | GSN;CAPG;FURIN;MMP8;MMP9;CAPNS1;ADAM15;CTSL;FLOT1;CTSG;CAPN1;LCP1;CD44;ELANE | 0.015 |
|  | GO:0022617 | extracellular matrix disassembly | GSN;CAPG;FURIN;MMP8;MMP9;CAPNS1;ADAM15;CTSL;FLOT1;CTSG;CAPN1;LCP1;CD44;ELANE | 0.015 |
|  | GO:0002281 | macrophage activation involved in immune response | GRN;SBNO2;DYSF;IFI35;NMI;LBP | 0.016 |
|  | GO:0061621 | canonical glycolysis | GPI;HK3;PKM;PGAM1;PGK1;ENO1;ALDOA;GAPDH | 0.016 |
|  | GO:0061718 | glucose catabolic process to pyruvate | GPI;HK3;PKM;PGAM1;PGK1;ENO1;ALDOA;GAPDH | 0.016 |
|  | GO:0032715 | negative regulation of interleukin-6 production | IL10;NLRP12;SYT11;GBA;ZC3H12A;SIRPA;PTPN22;NCKAP1L;BPI | 0.019 |
|  | GO:0061620 | glycolytic process through glucose-6-phosphate | GPI;HK3;PKM;PGAM1;PGK1;ENO1;ALDOA;GAPDH | 0.021 |
|  | GO:0038094 | Fc-gamma receptor signaling pathway | ACTR3;ACTR2;FCER1G;MYO10;ARPC1B;ARPC1A;PIK3CB;ARPC5;ACTB;ACTG1;FGR;HCK;ELMO2;NCKAP1L | 0.028 |
|  | GO:0030198 | extracellular matrix organization | COL17A1;ITGAM;LAMA2;COL14A1;LAMA1;CAPG;FURIN;ADAMTS12;ADAMTSL1;ADAMTS3;CAPNS1;MMP27;CTSL;ADAMTSL3;SPP1;FLOT1;CYP1B1;CTSG;QSOX1;SLC39A8;CAPN1;ELANE;COL28A1;GSN;LAMB3;COL23A1;MMP8;MMP9;COL1A1;ADAM15;GFOD2;COL21A1;LCP1;CD44;FBN1;PLEC | 0.030 |
|  | GO:0045055 | regulated exocytosis | TOR4A;CD63;SERPINA1;STXBP2;CLU;RIMS2;RIMS1;CYB5R1;SYNGR2;QSOX1;VTI1B;HGF;PCDH7;ANXA5;GTPBP2;F8;RAB31;SCCPDH;SYT11;DNAJC5;TLN1;CALM1;ALDOA;STX1A;FERMT3 | 0.030 |
|  | GO:0034123 | positive regulation of toll-like receptor signaling pathway | RSAD2;FLOT1;PTPN22;CYBA;IFI35;LBP;RTN4;LTF | 0.030 |
|  | GO:1902622 | regulation of neutrophil migration | BST1;RAC2;RHOG;NCKAP1L;OLFM4 | 0.030 |
|  | GO:0034314 | Arp2/3 complex-mediated actin nucleation | ACTR3;ACTR2;ARPC1B;ARPC1A;ARPC5;TRIM27 | 0.030 |
|  | GO:0002431 | Fc receptor mediated stimulatory signaling pathway | ACTR3;ACTR2;FCER1G;MYO10;ARPC1B;ARPC1A;PIK3CB;ARPC5;ACTB;ACTG1;FGR;HCK;ELMO2;NCKAP1L | 0.030 |
|  | GO:0030091 | protein repair | PCMT1;MSRB2;MSRB3;MSRB1 | 0.030 |
|  | GO:0043031 | negative regulation of macrophage activation | GRN;SYT11;ZC3H12A;BPI;CST7;LDLR | 0.040 |
|  | GO:0061025 | membrane fusion | NAPA;RIMS1;GCA;VAPA;MYOF;DYSF;CHP1;VTI1B;VAMP3 | 0.041 |
|  | GO:0098856 | intestinal lipid absorption | ABCG8;FABP2;UGCG;AKR1C1;LDLR | 0.041 |
|  | GO:0043097 | pyrimidine nucleoside salvage | CDA;TK2;UPP2;TK1;UPP1 | 0.041 |
|  | GO:0008655 | pyrimidine-containing compound salvage | CDA;TK2;UPP2;TK1;UPP1 | 0.041 |
|  | GO:0051156 | glucose 6-phosphate metabolic process | GPI;HK3;G6PD;TALDO1;PGM2;PGD;PGM2L1 | 0.047 |
|  | GO:0050765 | negative regulation of phagocytosis | SNX3;PLSCR1;SYT11;DYSF;SIRPA;PRTN3 | 0.049 |
|  | KEGG | Starch and sucrose metabolism | GPI;GYS1;HK3;UGP2;GBE1;AGL;PGM2;PYGL;GBA3;GYG1;PGM1;PGM2L1 | 3.84E-04 |
|  | KEGG | Salmonella infection | DCTN1;ARPC1B;ARPC1A;TXN;PIK3CB;NLRC4;ACTB;MYL12A;ACTG1;DYNC2LI1;ACTR1A;CASP7;TUBA1A;NCKAP1L;FLNB;KPNA4;MAP2K6;GSDMD;ACTR3;ACTR2;MLKL;DYNLT1;RHOG;ARPC5;MAPK14;SNX18;PLEKHM1;ELMO2;PFN1;GAPDH;NAIP;ARL8A | 0.019 |
|  | KEGG | Glycolysis/ Gluconeogenesis | GPI;ACSS2;PGAM1;ENO1;HK3;LDHA;PKM;ALDH3B1;PGK1;PGM2;ALDOA;GAPDH;PGM1 | 0.024 |
|  | KEGG | Bacterial invasion of epithelial cells | ACTR3;ACTR2;ARPC1B;ARPC1A;RHOG;PIK3CB;ARPC5;ACTB;ACTG1;CTNNA1;ELMO2;HCLS1;CTNNA3;CTNNA2 | 0.024 |
|  | KEGG | Pentose phosphate pathway | GPI;G6PD;PGM2;TALDO1;IDNK;PGD;ALDOA;PGM1 | 0.027 |

| Downregulated Pathways in Subclass 1 compared to Subclass 2 | GO:0042110 | T Cell Activation | ITK, CRTAM, CD3G, NLRC3, GATA3, CD3E, FOXP3, RASGRP1, LFNG, DPP4, CD2, ZAP70, CD4, LCK, CD7, CD28, TNFRSF4, ICOSLG, SOX4, ADA | 1.54 E -3 |
| --- | --- | --- | --- | --- |
|  | GO:0050853 | B Cell Receptor Signaling Pathway | CD79B, ITK, IGLL5, MEF2C, PLEKHA1, LCK, RFTN1, BLNK, BCL2, NFATC2, MS4A1 | 4.84E-3 |
|  | GO:0050851 | Antigen receptor-mediated signaling pathway | ITK, TXK, CD3G, GATA3, CD3E, CD3D, CD79B, BLNK, FYN, PLCG1, ICOSLG, MEF2C, PLEKHA1, TRAT1, RFTN1, THEMIS, NFATC2, ZAP70, IGLL5, CD4, PSMA1, LCK, BCL2, CD28, CD247, MS4A1, CARD11, SKAP1 | 7.26 E -3 |
|  | GO:0045580 | Regulation of T cell differentiation | CD2, ZAP70, TNFRSF18, CAMK4, IL2RA, TCF7, CRTAM, ZBTB1, NFATC2, GATA3, FOXP3 | 1.07 E-2 |
|  | GO:0042113 | B cell activation | CD86, TPD52, CD40, CR2, MEF2C, ITGA4, HDAC9, RASGRP1, CD79B, ZAP70, CD40LG, BANK1, BLNK, BCL2, MS4A1, CD22 | 0.028 |
|  | GO:0045059 | Positive thymic T cell selection | ZAP70, CD3G, CD3E, CD3D | 0.034 |
|  | KEGG | Primary immunodeficiency | ZAP70, CD40, CIITA, CD4, CD40LG, LCK, BLNK, CD3E, ICOS, IL7R, CD3D, ADA | 4.12E-04 |
|  | KEGG | T cell receptor signaling pathway | ITK, NFATC2, CBLB, CD3G, CD3E, RASGRP1, CD3D, VAV2, ZAP70, CD4, PPP3CC, CD40LG, LCK, CD28, FYN, PLCG1, CD247, ICOS, MAP3K14, CARD11 | 5.25E-04 |
|  | KEGG | Th17 cell differentiation | SMAD3, RORC, NFATC2, CD3G, GATA3, CD3E, FOXP3, CD3D, IL27RA, ZAP70, CD4, PPP3CC, IL23A, LCK, IL2RA, IL2RB, PLCG1, CD247 | 6.8 E -03 |
|  | KEGG | Th1 and Th2 cell differentiation | MAML2, NFATC2, CD3G, GATA3, CD3E, CD3D, RUNX3, ZAP70, CD4, PPP3CC, LCK, IL2RA, IL2RB, PLCG1, CD247 | 0.029 |

* The adjusted p-values are computed by using the Benjamini-Hochberg method for correction for multiple hypotheses testing.

Table E3: Differences in Whole Blood Cell Type Abundances between Subclass 1 and 2 using Cell Deconvolution

| **Cell Type** † | **Subclass 1** | **Subclass 2** | **p-value** |
| --- | --- | --- | --- |
| Fibroblasts | 1.4 ± 0.5 | 1.6 ± 0.4 | 0.18 |
| Endothelial Cells | 4.8 ± 0.7 | 4.2 ± 0.6 | 0.004* |
| Adipocytes | 2.6 ± 0.5 | 2.4 ± 0.5 | 0.005* |
| Keratinocytes | 1.3 ± 0.4 | 1.2 ± 0.3 | 0.46 |
| Schwann cells | 2.4 ± 0.4 | 2.3 ± 0.2 | 0.11 |
| Smooth muscle cells | 1.3 ± 0.6 | 1.0 ± 0.6 | 0.22 |
| CD34+ cells | 4.6 ± 1.7 | 3.7 ± 1.3 | 0.061 |
| Platelets | 6.7 ± 2.1 | 6.0 ± 2.3 | 0.28 |
| Monocytes- CD14+ CD16- | 2.6 ± 2.8 | 1.9 ± 2.9 | 0.41 |
| Monocytes- CD16+ CD14- | 3.2 ± 1.7 | 3.6 ± 1.9 | 0.57 |
| Macrophage | 7.1 ± 2.3 | 6.8 ± 1.6 | 0.56 |
| M1 (IFNgamma) | 1.1 ± 2.5 | 0.4 ± 1.3 | 0.20 |
| M2 (IL-4) | 3.1 ± 1.1 | 3.1 ± 1.2 | 0.96 |
| Dendritic cells (BDCA3+) | 3.0 ± 0.9 | 3.8 ± 1.0 | 0.007* |
| Dendritic cells (plasmacyte) | 5.0 ± 0.6 | 5.2 ± 0.8 | 0.26 |
| T cells (CD4+) | 0.9 ± 1.5 | 5.2 ± 4.3 | <0.001* |
| T cells (CD8+) | 1.7 ± 1.2 | 1.6 ± 0.9 | 0.79 |
| Natural Killer Cells | 4.4 ± 1.0 | 4.1 ± 1.4 | 0.51 |
| Neutrophils | 23.5 ± 4.6 | 22.8 ± 4.8 | 0.61 |
| B cells | 2.7 ± 2.0 | 4.6 ± 3.3 | 0.024* |

* Significant FDR values are noted with an asterisk.

†Data presented as mean percentage ± standard deviation

Table E4 Comparison of T Cell Repertoire between Subclass 1 and Subclass 2

|  |  | Total number of TCR reads † | p-value | Total number of TCR reads per 1 million RNA-Seq reads † | p-value |
| --- | --- | --- | --- | --- | --- |
| TRA | Subclass 1 | 181 (104 - 351) | 7.05e-05* | 3.92 (2.15 - 6.57) | 3.93 e-05* |
|  | Subclass 2 | 591 (360 - 695) |  | 10.51 (8.25 - 14.92) |  |
| TRB | Subclass 1 | 193 (104 - 298) | 0.0028* | 3.91 (2.38 - 6.31) | 0.0013* |
|  | Subclass 2 | 560 (331 - 968) |  | 10.04 (6.08 - 18.03) |  |
| TRD | Subclass 1 | 8 (6 - 13) | 0.029* | 0.18 (0.13 - 0.25) | 0.031* |
|  | Subclass 2 | 19 (7 - 49) |  | 0.37 (0.14 - 0.93) |  |
| TRG | Subclass 1 | 15 (7 - 19) | 0.10 | 0.29 (0.14 - 0.38) | 0.16 |
|  | Subclass 2 | 28 (7 - 39) |  | 0.49 (0.13 - 0.72) |  |

*Significant p values with p value <0.05 are noted with an asterisk.

† Data presented median (interquartile range)

Table E5 Comparison of Number of Clonotypes and Diversity between Subclass 1 and Subclass 2

|  |  | Number of clonotypes † | p-value | Inverse Simpson index † | p-value |
| --- | --- | --- | --- | --- | --- |
| TRA | Subclass 1 | 38 (30 - 65) | 0.00035* | 26.56 (21.44 - 44.36) | 0.0024* |
|  | Subclass 2 | 118 (80 - 173) |  | 85.38 (45.95 - 127.8) |  |
| TRB | Subclass 1 | 41 (28 - 66) | 0.0017* | 29.56 (17.17 - 49.35) | 0.002* |
|  | Subclass 2 | 114 (58 - 189) |  | 81.22 (39.54 - 137.28) |  |
| TRD | Subclass 1 | 2 (1 - 3) | 0.0285* | 1.92 (1 - 2.9) | 0.0359* |
|  | Subclass 2 | 4 (2 - 8) |  | 3.57 (1.96 - 6.6) |  |
| TRG | Subclass 1 | 3 (1 - 3) | 0.0479* | 4.45 (2.79 - 5.97) | 0.9688 |
|  | Subclass 2 | 4 (2 - 8) |  | 5.01 (1 - 14.54) |  |

*Significant p values with p value <0.05 are noted with an asterisk.

† Data presented median (interquartile range)

Table E6- Comparisons of Baseline Clinical Characteristics and Biomarkers across Controls and Subclasses

| **Measures** |  | **Controls** | **Subclass 1** | **Subclass 2** | **Three Group Comparison**  **FDR Adjusted P Value**† | **Subclass 1 versus Controls** | **Subclass 2 versus Controls** |
| --- | --- | --- | --- | --- | --- | --- | --- |
|  |  | **N = 52** | **N = 21** | **N = 25** |  |  |  |
| **Baseline Clinical Characteristics** |  |  |  |  |  |  |  |
|  | PRISM Score | 8 (3-14) | 14 (10-17) | 11 (5-19) | 0.013* | 0.004* | 0.259 |
|  | Average blood glucose (mg/dl) | 117 (100-148) | 134 (123, 170) | 116 (105, 125) | 0.013* | 0.012* | 0.999 |
| **Biomarkers** |  |  |  |  |  |  |  |
|  | PAI1 | 158 (102-360) | 500 (224-887) | 200 (94-308) | 0.002* | <0.0001* | 0.999 |
|  | IL4 | 9620 (3737-20797) | 7919 (3270-11897) | 4510 (1770-10910) | 0.179 | 0.857 | 0.114 |
|  | IL6 | 12 (7-23) | 148 (49-699) | 15 (9-52) | <0.0001* | <0.0001* | 0.205 |
|  | IL8 | 16 (11-23) | 34 (24-217) | 14 (13-20) | 0.001* | <0.0001* | 0.999 |
|  | IL10 | 9 (6-13) | 13 (10-72) | 10 (7-18) | 0.014 | 0.004* | 0.811 |
|  | ANG2 | 2608 (1764-4378) | 6936 (3255-8587) | 3414 (1901-6216) | 0.002* | <0.0001* | 0.666 |
|  | Thrombomodulin | 4357 (3246-5856) | 4891 (4234-651) | 4144 (3389 | 0.254 | 0.205 | 0.999 |
|  | TFPI | 11680 (8376-26687) | 12027 (7262-18706 | 13440 (7036-17308) | 0.520 | 0.929 | 0.498 |
|  | TREM1 | 289 (218,6-381.2) | 489 (330-578) | 325 (258-427) | 0.003* | 0.001* | 0.655 |
|  | P-selectin | 5769 (4256-9144) | 6414 (4880-8740) | 6878 (4623-8885) | 0.769 | 0.999 | 0.999 |
|  | ICAM1 | 63701 (42301-86131) | 130000 (97558-193000) | 113000 (80346-164000) | <0.0001* | <0.0001* | 0.000* |
|  | TNFR | 797 (543-1086( | 1833 (1466-2284) | 1106 (932-1296) | <0.0001* | <0.0001* | 0.041* |

Data presented as median (IQR)

*Significant FDR values are noted with an asterisk.

†P-values computed by Wilcoxon rank sum test and adjusted by FDR criterion
